# Testing a Novel Deliberate Practice Intervention to Improve Diagnostic Reasoning in Trauma Triage: A Pilot Randomized Clinical Trial

**DOI:** 10.1001/jamanetworkopen.2023.13569

**Published:** 2023-05-17

**Authors:** Deepika Mohan, Jonathan Elmer, Robert M. Arnold, Raquel M. Forsythe, Baruch Fischhoff, Kimberly Rak, Jaqueline L. Barnes, Douglas B. White

**Affiliations:** 1 Department of Surgery, University of Pittsburgh School of Medicine, Pittsburgh, Pennsylvania; 2 Department of Critical Care Medicine, University of Pittsburgh School of Medicine, Pittsburgh, Pennsylvania; 3 Department of Emergency Medicine, University of Pittsburgh School of Medicine, Pittsburgh, Pennsylvania; 4 Department of Neurology, University of Pittsburgh School of Medicine, Pittsburgh, Pennsylvania; 5 Division of Palliative Care, Department of Medicine, University of Pittsburgh School of Medicine, Pittsburgh, Pennsylvania; 6 Department of Engineering and Environmental Policy, Carnegie Mellon University, Pittsburgh, Pennsylvania

## Abstract

**Importance:**

Diagnostic errors made during triage at nontrauma centers contribute to preventable morbidity and mortality after injury.

**Objective:**

To test the feasibility, acceptability, and preliminary effect of a novel deliberate practice intervention to improve diagnostic reasoning in trauma triage.

**Design, Setting, and Participants:**

This pilot randomized clinical trial was conducted online in a national convenience sample of 72 emergency physicians between January 1 and March 31, 2022, without follow-up.

**Interventions:**

Participants were randomly assigned to receive either usual care (ie, passive control) or a deliberate practice intervention, consisting of 3 weekly, 30-minute, video-conferenced sessions during which physicians played a customized, theory-based video game while being observed by content experts (coaches) who provided immediate, personalized feedback on diagnostic reasoning.

**Main Outcomes and Measures:**

Using the Proctor framework of outcomes for implementation research, the feasibility, fidelity, acceptability, adoption, and appropriateness of the intervention was assessed by reviewing videos of the coaching sessions and conducting debriefing interviews with participants. A validated online simulation was used to assess the intervention’s effect on behavior, and triage among control and intervention physicians was compared using mixed-effects logistic regression. Implementation outcomes were analyzed using an intention-to-treat approach, but participants who did not use the simulation were excluded from the efficacy analysis.

**Results:**

The study enrolled 72 physicians (mean [SD] age, 43.3 [9.4] years; 44 men [61%]) but limited registration of physicians in the intervention group to 30 because of the availability of the coaches. Physicians worked in 20 states; 62 (86%) were board certified in emergency medicine. The intervention was delivered with high fidelity, with 28 of 30 physicians (93%) completing 3 coaching sessions and with coaches delivering 95% of session components (642 of 674). A total of 21 of 36 physicians (58%) in the control group participated in outcome assessment; 28 of 30 physicians (93%) in the intervention group participated in semistructured interviews, and 26 of 30 physicians (87%) in the intervention group participated in outcome assessment. Most physicians in the intervention group (93% [26 of 28]) described the sessions as entertaining and valuable; most (88% [22 of 25]) affirmed the intention to adopt the principles discussed. Suggestions for refinement included providing more time with the coach and addressing contextual barriers to triage. During the simulation, the triage decisions of physicians in the intervention group were more likely to adhere to clinical practice guidelines than those in the control group (odds ratio; 13.8, 95% CI, 2.8-69.6; *P* = .001).

**Conclusions and Relevance:**

In this pilot randomized clinical trial, coaching was feasible and acceptable and had a large effect on simulated trauma triage decisions, setting the stage for a phase 3 trial.

**Trial Registration:**

ClinicalTrials.gov Identifier: NCT05168579

## Key Points

**Question:**

Can deliberate practice (goal-oriented training with a coach who provides immediate, personalized performance feedback) improve diagnostic reasoning in trauma triage?

**Findings:**

In this pilot randomized clinical trial of a novel deliberate practice intervention, 93% of participants received 3 planned coaching sessions, and most participants (93%) described the sessions as entertaining and valuable. During a simulation, the triage decisions of physicians in the intervention group were more likely to adhere to clinical practice guidelines than the triage decisions of physicians in the control group.

**Meaning:**

The deliberate practice intervention was feasible, acceptable, and effective in the laboratory, setting the stage for a future phase 3 clinical trial.

## Teaser

This pilot randomized clinical trial conducted among emergency physicians tests the feasibility, acceptability, and preliminary effect of a novel deliberate practice intervention to improve diagnostic reasoning in trauma triage.

## Introduction

Half of all injured patients present initially to a nontrauma center, where a clinician must evaluate and stabilize the patient’s injuries and determine whether they warrant transfer to a trauma center.^1,2^ Timely and guideline-concordant referral reduces mortality by 10% to 25%, increases rates of functional independence, and shortens the duration of pain and disability.^3,4,5,6,7,8,9^ Despite 40 years of efforts by stakeholders to standardize triage practices, undertriage remains common, particularly among patients older than 65 years.^10,11,12,13^ Diagnostic errors—defined as the failure to establish an accurate and timely explanation of the patient’s health problem—are an important cause of undertriage.^14,15^

Deliberate practice, defined as goal-oriented training in the presence of a content expert who can provide personalized, immediate feedback to improve performance, has successfully improved outcomes across multiple domains, including sports, combat, and surgery.^16,17,18^ However, the use of deliberate practice to improve diagnostic reasoning is uncommon and, to our knowledge, has never been tried in trauma triage.^19^

The objective of this pilot randomized clinical trial was to test the feasibility (practicability), fidelity (delivery of tasks), acceptability (palatability), adoption (intention to try behaviors), appropriateness (fitting the user’s goals and needs), and effect (compliance with clinical guidelines) of a novel deliberate practice intervention in trauma triage.

## Methods

### Study Overview

We conducted a pilot randomized clinical trial of a deliberate practice intervention to improve diagnostic reasoning in trauma triage between January 1 and March 31, 2022, without follow-up. We enrolled and randomized a national respondent-driven sample of physicians to the intervention group or to a passive control group. We structured the process evaluation of the intervention using the Proctor framework of outcomes for implementation research and followed the Consolidated Standards of Reporting Trials Extension (CONSORT Extension) reporting guideline (ie, extension for pilot and feasibility trials) in reporting our results.^20,21^ We previously published the trial protocol with a priori hypotheses about criteria for defining success.^22^ The University of Pittsburgh Human Research Protection Office approved the study. Trial participants provided digital written informed consent at the time of enrollment (trial protocol in Supplement 1).

### Trial Participants and Coaches

**Figure 1.**
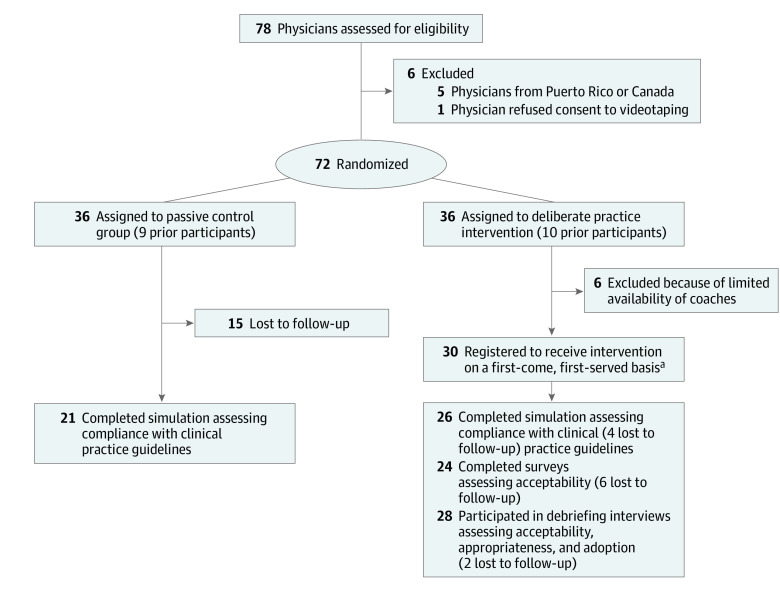
Flow Diagram Description of participant enrollment, allocation, and analysis. ^a^The tasks were not mutually exclusive—we wanted the 30 physicians to do all 3 tasks.

To recruit participants for the study, we contacted physicians who had previously participated in our research and asked them to refer us to 2 colleagues. We sought board-certified emergency physicians who treated adult patients in the emergency department of either a nontrauma center or a Level III or IV trauma center in the US and who therefore would have responsibility for performing trauma triage in their clinical practice. Respondents received a screening questionnaire with details about the trial, a consent form, and items querying their demographic characteristics. Racial and ethnic categories were specified by the study team based on National Institutes of Health criteria.^23^ Physicians who provided consent were randomized in a 1:1 ratio, stratified by prior participation in our research, using a schema built in Stata, version 16.0 (StataCorp LLC), with block sizes of 4 (Figure 1). Although we could not blind study personnel and participants, we masked physicians’ exposure during analysis.

Three members of the study team with expertise in trauma surgery (D.M. and R.M.F.) and emergency medicine (J.E.) acted as the coaches. We standardized the fidelity of intervention delivery in 3 ways. First, prior to the trial, we conducted three 1-hour training sessions, supervised by experts in deliberate practice (R.M.A., B.F., and D.B.W.). Second, we created a coaching manual as a reference that summarized the learning objectives, core tasks of the coaching sessions, and the pedagogical strategies that coaches should use (a full draft of the coaching manual is in the eAppendix in Supplement 2). Finally, coaches met weekly with the full study team during the trial to debrief and to discuss strategies for managing issues that had arisen. Based on these sessions, we made several modifications to the intervention, including condensing the content to increase the time spent on each decision principle and identifying additional pedagogical strategies that coaches could use to engage participants in the sessions (eg, retrieval practice during sessions 2 and 3).

### Interventions

#### Deliberate Practice

**Figure 2.**
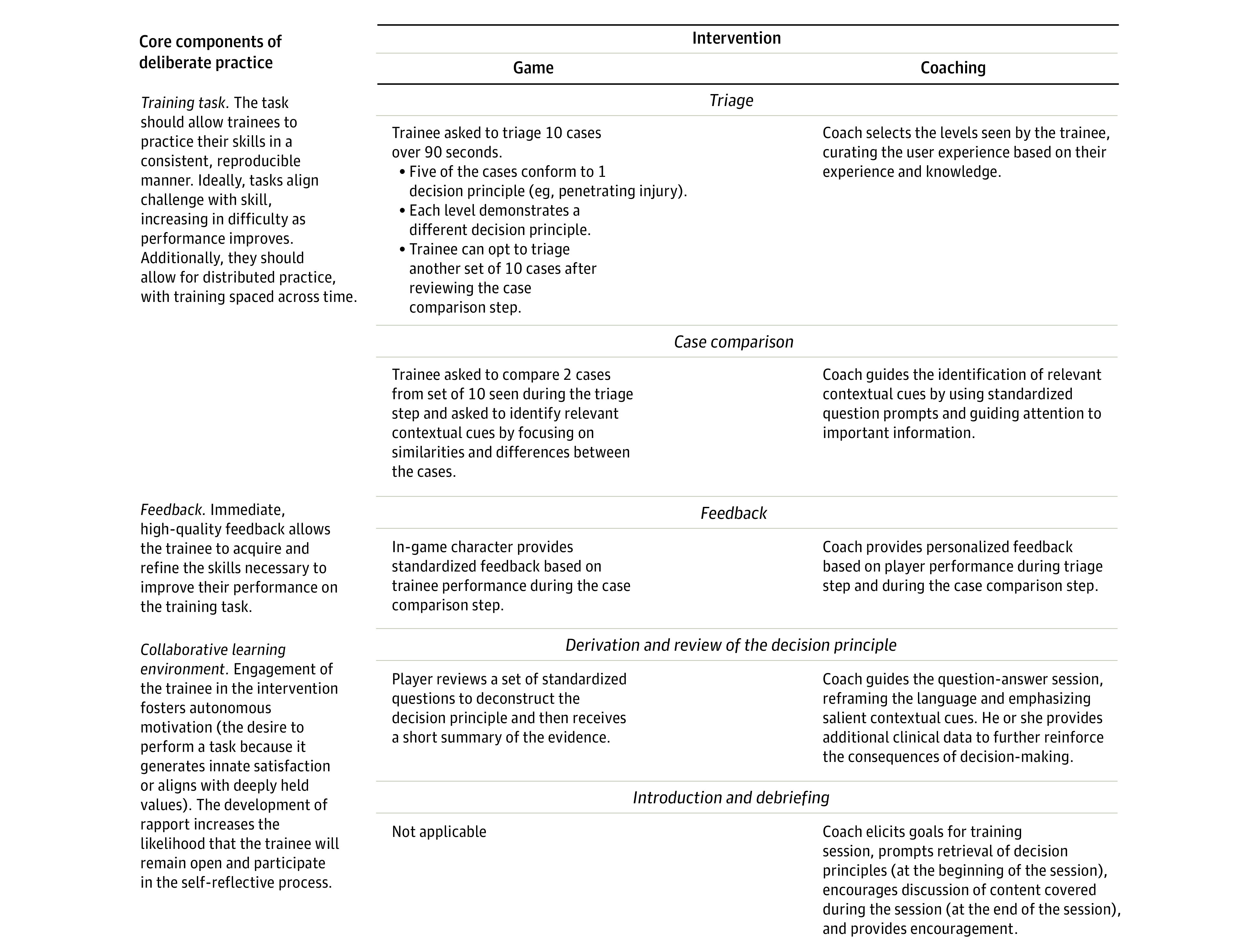
Conceptual Framework of Intervention

The intervention consisted of 3 weekly, 30-minute, video-conferenced coaching sessions, in which the participant played a trauma triage video game, the coach observed his or her performance, and they discussed best practice decision principles in trauma triage. We describe the conceptual framework of the intervention in Figure 2.

##### Video Game

We used a single-player, theory-based puzzle video game, previously developed by our group to improve diagnostic reasoning in trauma triage (*Shift: The Next Generation*).^24^ To allow its use as a training task, we adapted the user interface and game mechanics in collaboration with Schell Games, creating *Shift With Friends*. The game included 10 levels, each covering a separate decision principle and involving a 5-step game loop (eFigure in Supplement 2): players triaged 10 injured patients over 90 seconds, compared 2 cases to identify similarities or differences so that they could derive the rule for the level, received standardized feedback on their performance, reviewed the decision principle, and finally received a synthesis of the evidence supporting the decision principle.

##### Coaching

Both the participant and the coach logged into Zoom, and the participant shared his or her screen so that the coach could observe gameplay. The coach would select the levels covered during the session, personalizing the selection to the needs and skills of the participant. The coach would also encourage the participant to “think aloud” as he or she played, using observations made during the process to provide feedback tailored to improve the participant’s diagnostic reasoning. Each session covered 1 to 3 decision principles and included 6 to 8 tasks (eg, introductions or debriefing).

#### Passive Control

We did not ask trial participants randomly assigned to the control group to engage in any additional continuing medical education, with the intention of replicating usual care.

### Trial Protocol

After randomization, participating physicians received written instructions on how to complete the trial tasks. We had the capacity to provide coaching for 30 physicians. We therefore asked those in the intervention group to select 1 of the 2 blocks (January or February) in which we offered coaching and to sign up for three 30-minute sessions within the block. Based on availability, we paired participants with a coach on a first-come, first-served basis. After the sessions, we asked participants to complete a survey, a semistructured debriefing interview, and an online simulation. We asked participants in the passive control group to complete the same simulation within 3 weeks of the start of the trial. The trial tasks took approximately 3 hours for those in the intervention group and 1 hour for those in the control group. Participants received 3 personalized reminder emails at weekly intervals or until they completed the trial tasks. We offered a financial incentive to increase response rates, setting its size with a wage-based model of reimbursement.^25,26^ Physicians in the intervention group received an iPad with the game and Zoom app preloaded, which they used for the coaching sessions and which they kept as their honorarium (approximate value, $300). Those in the control group received a $100 gift card after they completed the simulation.

### Outcomes

Using the Proctor framework of outcomes for implementation research, we assessed both implementation and service outcomes.^20^ We defined the implementation outcomes as feasibility, fidelity, acceptability, adoption, and appropriateness. Using the National Institutes of Health stage model of intervention development, which recommends assessment of efficacy in the laboratory before moving to real-world testing,^27^ we defined the service outcome (efficacy) as compliance with clinical practice guidelines, measured using a simulation.

### Data Sources and Management

#### Screening Questionnaire and Tracking Database

Each respondent described his or her personal characteristics on the screening questionnaire at the time of enrollment. We maintained a database with a list of scheduled coaching sessions, which was updated daily with the status of the sessions.

#### Coaching Sessions

We recorded all the coaching sessions and automatically uploaded them to a secure server hosted by the University of Pittsburgh. Two members of the study team (K.R. and J.L.B.) developed a codebook to assess the delivery of session tasks, refined it until they achieved acceptable interrater reliability (Cohen κ = 0.84), and independently applied it to the recordings. Coding discrepancies were resolved through consensus (D.M., K.R., and J.L.B.). We used NVivo qualitative analysis software (QSR International) for data management.

#### Postintervention Debriefing Materials

Participants in the intervention group provided structured assessments of the acceptability of the intervention using the User Engagement Scale–Short Form to evaluate the video game (a validated 12-item instrument with a 5-point Likert scale) and the Wisconsin Surgical Coaching Rubric to evaluate the quality of the coaching (a 4-item instrument with a 5-point scale).^28,29^ They also participated in semistructured debriefing interviews after the final coaching session, during which they discussed their perception of the acceptability, adoption, and appropriateness of the intervention. Two members of the study team (K.R. and J.L.B.) coded the interviews using the same process as for the coaching sessions (Cohen κ = 0.84).

#### Simulation to Measure Efficacy

We used a validated 2-dimensional simulation to assess compliance with guidelines after exposure to the intervention.^30^ The simulation required participants to respond to 10 cases over 42 minutes: 4 severely injured patients, 2 minimally injured patients, and 4 critically ill nontrauma patients (ie, distractor patients). New patients arrived at prespecified but unpredictable intervals, so that users managed multiple patients concurrently. Without clinical intervention by the player, severely injured patients and critically ill distractor patients decompensated and died over the course of the simulation. Each case included a 2-dimensional rendering of the patient, a chief symptom, vital signs that updated every 30 seconds, a history, and a written description of the physical examination. Users could request information by selecting from a prespecified list of 250 medications, studies, and procedures. They could place orders and request consultations. Each case ended when either the player made a disposition decision (admit, discharge, or transfer) or the patient died. We asked all trial participants to complete the simulation online; responses were uploaded and stored on a secure server hosted by the University of Pittsburgh.

### Statistical Analysis

We summarized physician characteristics using mean (SD) values for continuous variables and counts and percentages for categorical variables. We analyzed implementation outcomes using an intention-to-treat approach but excluded from the efficacy analysis participants who did not use the simulation. We had 2 criteria for the success of the trial: efficacy and feasibility. Our primary hypothesis was that physicians exposed to the intervention would undertriage 25% fewer patients or more on the simulation than physicians in the control group. All *P* values were from 2-sided tests and results were deemed statistically significant at *P* < .05. Our secondary hypothesis was that we could deliver 3 coaching sessions to 90% or more of participants. All analyses were conducted in Stata, version 16.0 (StataCorp LLC).

#### Implementation Outcomes

We quantified the percentage of coach-participant dyads that completed three 30-minute sessions (to measure feasibility) and summarized the percentage of session tasks delivered to participants (to measure fidelity). We summarized participant responses to the User Engagement Scale–Short Form and to the Wisconsin Surgical Coaching Rubric (to measure acceptability). We also summarized themes that arose during the semistructured interviews (to further assess acceptability and to assess appropriateness and adoption).

#### Efficacy

We summarized the time spent and the decisions made for each severely injured trauma case (n = 4) on the simulation (eg, diagnostic testing or administration of blood products) using median values and IQRs, and we scored disposition decisions as consistent with the American College of Surgeons guidelines or not. To compare differences between the intervention and control groups, we fit a mixed-effects logistic regression model, clustered at the participant level, with the transfer decision as the dependent variable and physicians’ exposure as the primary independent variable. Given the statistical power, we did not adjust for any potential confounders (eg, practice environment). In a post hoc sensitivity analysis, we excluded physicians who had previously participated in our research.

#### Human Participants and Power Calculation

We designed the experiment to detect a 25% (large effect size) reduction in undertriage between physicians in the intervention and control groups, with an α of .05 and a power of 80%, using the Cohen method of estimating power for behavioral trials. Based on these estimates, and anticipating a 67% retention rate in the control group, we planned to recruit 30 physicians for each group.^31^

## Results

### Participant Characteristics

We randomly assigned 72 physicians to the 2 groups of the trial but limited registration of physicians in the intervention group to 30 because of the availability of the coaches (Figure 1). Physicians were mostly middle aged (mean [SD], age, 43.3 [9.4] years]), male (44 [61%]), White (47 [65%]), and board-certified in emergency medicine (62 [86%]) and had completed Advanced Trauma Life Support certification (64 [89%]) (Table 1). They lived in 20 states, primarily in the Northeast and Southeast (38 [53%])

**Table 1.** Participant Characteristics

Characteristic	Participants, No. (%) (N = 72)
Age, mean (SD), y	43.3 (9.4)
Gender	
Male	44 (61)
Female	28 (39)
Race and ethnicity	
African American or Black	3 (4)
American Indian or Alaska Native	1 (1)
Asian	17 (24)
Hispanic or Latino or Latina	4 (6)
White	47 (65)
ATLS certification–yes	64 (89)
Specialty	
Emergency medicine	62 (86)
Family practice	4 (6)
Internal medicine	3 (4)
Other (eg, general surgery)	3 (4)
Region	
Northeast	19 (26)
Southeast	19 (26)
Midwest	9 (13)
Southwest	8 (11)
West	17 (24)

Abbreviation: ATLS, Advanced Trauma Life Support.

Of the 36 physicians in the control group, 21 (58%) finished the virtual simulation. The characteristics of the responders and nonresponders are listed in eTable 1 in Supplement 2. Of the 30 physicians registered to receive the intervention, 24 (80%) responded to the survey, 28 (93%) participated in the debriefing interviews, and 26 (87%) used the virtual simulation.

### Feasibility and Fidelity

We summarize our assessment of the intervention in Table 2. Most physicians (28 of 30 [93%]) completed 3 coaching sessions; 2 of 30 (7%) completed 2 sessions. The sessions lasted a mean of 31.2 (0.2) minutes; participants covered a mean (SD) of 5.2 (1.1) decision principles during the 3 sessions. Coaches covered 95% of the tasks (642 of 674), pooled across the 3 sessions. The component most frequently missed was the debriefing (19% [6 of 32]), usually because of time constraints.

**Table 2.** Summary of Assessment of Intervention Using the Proctor Framework of Outcomes in Implementation Research

Outcome	Definition	Status, No./total No. (%)
**Implementation^a^**		
Feasibility: practicability of delivering the intervention as planned	Percentage of coach-trainee dyads that completed all 3 training sessions	28/30 (93)
Fidelity: completion of tasks listed in the coaching manual	Percentage of session tasks delivered to each participant, summed across the total number of sessions completed	642/674 (95)
Acceptability: perception that the intervention is agreeable, palatable, or satisfactory	Percentage of intervention participants who described sessions as valuable	26/28 (93)
Adoption: intention to try the intervention	Percentage of intervention participants who stated the intention to use the principles encoded in the intervention	22/25 (88)
Appropriateness: perceived fit of the intervention	Percentage of intervention participants who described the length and number of sessions as appropriate	16/20 (80)
**Service^b^**		
Efficacy: compliance with clinical practice guidelines for the triage of patients with trauma	Increase in compliance between physicians in the intervention and control groups on a virtual simulation	36^c^

^a^ A prespecified criterion for the success of the trial was to have 90% or more of dyads complete all 3 training sessions.

^b^ A prespecified criterion for the success of the trial was to have a 25% reduction in undertriage.

^c^ This represents the difference in compliance between the 2 groups: 50 of 99 for the intervention group and 13 of 84 for the control group.

### Acceptability, Appropriateness, and Adoption

In semistructured interviews, most participants (93% [26 of 28]) in the intervention group described the sessions as entertaining, providing a useful refresher of guidelines, distilling clear learning points, and modeling valuable communication scripts for emergency department physicians. Most participants responded that the length and number of sessions were appropriate (80% [16 of 20]) and would recommend the intervention (87% [20 of 23]). Of the 25 physicians who discussed adoption of the principles, 6 (24%) reported having used the material since completing the coaching sessions, while 16 (64%) said they would use the material in the future. Some participants (7 of 28 [25%]) had reservations about the program. For example, 1 participant noted a discordance between the intervention and the realities of clinical practice; another responded that the time commitment was excessive. We provide additional qualitative assessments of the intervention by participants in the Box.

Box. **Participant Assessments of the Acceptability, Appropriateness, and Adoption of the Intervention During Semistructured Interviews**

**Acceptability**

**Theme: intervention valuable (26 of 28 [93%])**

Subtopic: entertaining and fun
**Sample quotations:**
    “I mean I personally liked it a lot, so I was actually looking forward to doing the next session. The cases were fun to do and still at the end the review allowed you to sort of put it together, and get better at it actually, for the next time.”
    “I’d never done anything like that before in any of my training. But I liked that it felt like a game, and I liked getting the feedback from the person instantly through the coaching. I thought that was definitely, that’s how I learn best, getting some feedback right away.”
    “It was a good way of engaging and teaching information because I think I’ve been on Zoom the past year and a half listening to lectures and no one’s listening. So, this was for the you know adult learners who you know need kind of more than just listening to someone talk and look at the same slides you know it’s a much better way to learn.”
Subtopic: a useful refresher of guidelines
**Sample quotations:**
    “Yeah, I think it’s perfect. You know I think we as physicians we feel like, ‘Oh you know I’m going through training I know everything’ but we know that we forget.”
    “I think they were helpful. I realized that maybe some of my decisions that I make at work are not the best, like sometimes I’ll choose not to transfer patients when according to the simulation and according to you know speaking to the surgeon, I know they should be, even though I have the resources available and so that was really good to point that out to me. So, I think it’s actually going to change my practice a little bit.”
Subtopic: provides evidence that underlies practice; distills clear learning point
**Sample quotation:**
    “I know when to transfer and I know when not to but then you know kind of distilling it into what is it exactly about this patient, what are the reasons we make that decision. I guess it’s not like I sit and think about it. It was just kind of like that’s what we always do or that’s what we know what we usually do but this kind of like helped me kind of clarify in my mind what those criteria are. And I thought you know for someone [who] had no experience at a trauma center and they were going to go work somewhere that it’ll be a great way for them too especially at the resident level as well, but even anyone that was just changing their practice environment, using that too, it was a good way of engaging and teaching information…”
Subtopic: provides language to discuss cases with consultant services
**Sample quotation:**
    “I found [the sessions] valuable. They helped concretize some principles that I think I will use next time I’m actually speaking to somebody on the other end of the phone. And [coach] had a nice suggestion about how to, you know, craft that conversation. Because it’s a frustrating piece of our jobs, trying to get folks accepted and sort of make the right pitch to our person. And so, I think this was helpful for me to refine that language. You know, even though, I’ve been doing it [xx] years I feel like I’ve refined it quite a lot, but there’s always opportunity to get it a little bit better.”
Subtopic: provides an opportunity to potentially improve patient outcomes
**Sample quotation:**
    “I think if you know if other people can kind of pick up some of these learning lessons, more people will survive these traumas, so. Especially if you can somehow target like the critical access hospitals where there’s only like 1 or 2 docs, those are the places where if they can kind of, the lessons that the game has to teach [inaudible] I think it’ll save a lot of lives…”

**Theme: intervention not valuable (7 of 28 [25%])**

Subtopic: does not reflect realities of clinical practice
**Sample quotation:**
    “You’re asking me to transfer a patient out based on let’s say their age or injury, and you know a lot of the times the surgeon in house, if I’m working in a small hospital, I only know what they can handle and cannot handle, and it’s not totally up to me to transfer someone out. If I call a surgeon and they would say, ‘Hey I can take care of that, please admit to my service.’ Defying that has some repercussions if you’re working in a small hospital, you cannot just say I work independently. Nobody does. Right? You work as part of a hospital or part of a group or team. So, there’s lots of gray lines, sometimes somebody says I can take care of, or I cannot take care of, and you based on the rest of the team. Right, this game almost made it seem like it’s not a team, you’re making the decision and I don’t think that’s true. So, it made it very black and white and that’s not true.”
Subtopic: rudimentary
**Sample quotation:**
    “You know, a number of the concepts the app is designed to teach or reinforce were those that I consider myself reasonably well familiar with. So, from that standpoint, I’m not sure I learned a ton, although I can certainly see its utility for other providers.”

**Appropriateness**

**Theme: time burden (20 of 28 [71%])**

Subtopic: participants responded that the length and number of sessions were appropriate (16 of 20 [80%])
**Sample quotation:**
    “They were great, [coach] was wonderful, the game was fun. They were just the right amount of length, you know what I mean we did like a I think a half an hour 3 times it was like, we got to play like 1 or 2 games each time it wasn’t like it got like, it wasn’t like too long I suppose. Enough to keep you interested.”
Subtopic: participants wanted more time with the coach (3 of 20 [15%])
**Sample quotation:**
    “Again, I think as I said, the length of the session I think should be a little bit longer maybe. Just when you start to feel comfortable, they’re like, ‘Okay that’s it.’ And I know it’s hard for any doctors to get together for more than any tiny period of time, but I think the process was fulfilling and might’ve been even more so with longer sessions.”
Subtopic: participants responded that the time commitment was excessive (1 of 20 [5%])
**Sample quotation:**
    “I think so I think it’s like you know worth the experience…it can be a little bit more streamlined, and you know hour and a half seems a lot of time for that…”

**Theme: would recommend (23 of 28 [82%])**

Subtopic: unreservedly (20 of 23 [87%])
**Sample quotation:**
    “I didn’t know what this was going to be frankly…when I signed up; but I was pleasantly surprised. I think it was very engaging. I almost would, you know if this was available, I would probably use it for my own doctors at my department. I think people would find it very useful. So very pleasantly surprised and very happy to participate in this to the end.”
Suptopic: for others or with some changes (3 of 23 [13%])
**Sample quotation:**
    “I think there’s probably a zone in between where I work (where every trauma gets transferred) and others (where there are criteria for what to transfer) where you get the most value…”

**Adoption**

**Theme: adoption (25 of 28 [89%])**

Subtopic: have already done so (6 of 25 [24%])
**Sample quotations:**
    “Yeah, so I work with residents. So, although it was useful to me, you know, every time like I do something and like something I just learned comes up I kind of pass it on to somebody else. They’re like, ‘Oh why are we thinking about transferring him,’ ‘Oh it’s because he’s frail’ or ‘Oh it’s because you know he has 2 organs injured’ or you know something like that.”
    “Yeah, I worked 60 hours the last few days in the community setting and had had several trauma patients that I thought about these sessions and what the best course of care would be, and it actually really is kind of impactful I think.”
Subtopic: plan to do so in the future (16 of 25 [64%])
**Sample quotation:**
    “Yeah, I think I will be able to, yeah definitely. Because we see trauma every day, minor trauma, some major which we have to transfer…”
Subtopic: not specifically (3 of 25 [12%])
**Sample quotation:**
    “Not specifically like I said I think there are some general learning concepts that were confirmatory for me, again I think they were fairly basic in nature so certainly it is valuable because it confirms what someone already knows and I think for some it will be again very valuable and into the theme would be, again something new for them.”

Responses to the surveys also were positive. For example, 96% (23 of 24) agreed or strongly agreed that their experience with the game was worthwhile, and 100% (24 of 24) strongly agreed that the coach provided constructive feedback. We provide complete responses to the surveys in eTable 2 in Supplement 2.

### Efficacy

Physicians in the intervention group spent a median of 5.3 minutes (IQR, 3.6-7.7 minutes) on severely injured cases and entered a median of 10 orders per case (IQR, 8-13 orders per case), while those in the control group spent a median of 6.8 minutes (IQR, 4.8-9.2 minutes) and entered a median of 11 orders per case (IQR, 9.5-15.0 orders per case). Physicians in the intervention group were more likely than physicians in the control group to transfer severely injured patients to trauma centers (51% [50 of 99] vs 15% [13 of 84]; odds ratio, 13.8; 95% CI 2.8-69.6; *P* = .001). The effect of the intervention remained after excluding the 12 physicians who had used the simulation in prior research, participated in this trial, and completed trial tasks (57% [43 of 76] vs 17% [11 of 64]; odds ratio, 16.3; 95% CI, 2.8-94.3; *P* = .002).

## Discussion

In this pilot randomized clinical trial, we delivered a novel deliberate practice intervention to practicing emergency medicine physicians with high fidelity. Most physicians described the intervention as valuable and the time required as appropriate. They also reported intentions to adopt the lessons learned during the training sessions. The intervention had an effect on physicians’ adherence to trauma triage practice guidelines during an online simulation.

These results confirm those of previous studies, which demonstrated the ability of deliberate practice to facilitate the acquisition of expertise in medicine, across domains as varied as communication in the emergency department or intensive care unit, resuscitation, and surgery.^32,33,34,35,36^ For example, a meta-analytic comparative review of the literature on skill acquisition in procedural domains (eg, laparoscopic surgery) identified a correlation between the use of deliberate practice and positive outcomes (*r* = 0.71).^32^ Our study expands on these results by focusing on diagnostic reasoning and targeting the performance of physicians in practice rather than medical trainees.

If these results transfer to the clinical setting, deliberate practice could address national priorities to improve public health outcomes.^14,37^ Injury is the leading cause of loss of life among people younger than 45 years and the leading cause of loss of independence among people older than 65 years.^38^ More generally, diagnostic errors affect between 8% and 15% of all hospital admissions in the US, with cognitive factors in clinician decision-making contributing to 75% of the errors.^39,40^

This pilot randomized clinical trial allowed us to test several concerns about delivering a deliberate practice intervention in trauma triage. One concern was how to design a training task that captured the core challenges of trauma triage at nontrauma centers—uncertainty, time pressure, and low rates of true-positive cases. The use of a customized video game provided physicians with an opportunity to practice the diagnostic task repeatedly and rapidly. A second concern was how to help participants refine the heuristics (intuitive judgments) guiding their judgments. Having participants think aloud while playing the game gave coaches access to participants’ cognitive processes, expressed in their natural terms, helping coaches to select and to convey potentially missed cues (eg, frailty). A third concern was the feasibility and acceptability of such an intervention among busy practicing clinicians. We found that most physicians enrolled in the trial, albeit a self-selected sample, relished the opportunity to interact with someone whom they perceived as a content expert. In debriefing interviews, respondents emphasized the social aspect of the intervention, underscoring the value they found in the connections that they formed with the coach.

### Limitations

This study has several limitations. First, we used a passive rather than an active control as the comparator, which may have magnified the effect of the intervention. Second, attrition in the completion of the virtual simulation differed among the control and intervention groups, which we attribute to engagement. Attrition could have introduced bias, if missingness was not random. However, we have no reason to believe that this was the case. Third, our observations may lack generalizability because of the characteristics of our convenience sample. Fourth, we did not cluster our analysis at the coach level during this pilot trial but plan to test this potential mediator of effect in the future.

## Conclusions

This successful pilot randomized clinical trial sets the stage for a planned phase 3 trial of our novel behavioral intervention. The trial also provides evidence that deliberate practice can support judgment tasks, as well as the procedural tasks addressed in most applications, and therefore extends the utility of the method.

## Notes

Supplement 1.
Trial Protocol

Supplement 2.
eAppendix.

eReferences.
**eFigure.** Screenshots of Game Loop **eTable 1.** Characteristics of Responders and Nonresponders to the Virtual Simulation **eTable 2.** Structured Assessment of Acceptability of Intervention

Supplement 3.
Data Sharing Statement
