# Supplementary material for: Testing a Novel Deliberate Practice Intervention to Improve Diagnostic Reasoning in Trauma Triage: A Pilot Randomized Clinical Trial
Source: JAMA Netw Open. 2023 May 17;6(5):e2313569. doi: 10.1001/jamanetworkopen.2023.13569 (PMC10193186; doi:10.1001/jamanetworkopen.2023.13569)
Supplement: Supplement 1. — Trial Protocol [file jamanetwopen-e2313569-s001.pdf]

PittPRO Pitt Protocol Review Online

Date: Wednesday, November 30, 2022 11:58:07 AM

Print Close

View: Pitt SF: Basic Study Information 8.2

Basic Study Information

1. * Title of study:

Developing a deliberate practice intervention to recalibrate physician heuristics in trauma triage

2. * Short title:

Feasibility and efficacy of a deliberate practice intervention in trauma triage.

3. * Brief description:

The objective of this study is to test the feasibility of using deliberate practice – goal-oriented training in the presence of a coach who can provide personalized, immediate feedback – to increase engagement. The research design involves recruitment of a national convenience sample of board-certified emergency physicians who will serve as trainees (n=30), pairing of the trainees with a coach, delivery of three 30-minute coaching sessions using the existing games as the training task, and assessment of the effect of the combined intervention on performance in the laboratory. The specific aims are:

1. To assess the fidelity of intervention delivery by measuring coaching skill acquisition, coaching skill drift and protocol adherence.
2. To assess the potential effect size of the intervention by comparing trainee performance on a validated virtual simulation with a control group of physicians (n=30).
3. To assess the acceptability of the intervention by using a mixture of validated instruments and semi-structured debriefing interviews with trainees to assess their engagement with the intervention.

4. * What kind of study is this?

Single-site study

5. * Will an external IRB act as the IRB of record for this study?

○ Yes ● **No**

6. * Local principal investigator:

Deepika Mohan

*** Is this your first submission, as PI, to the Pitt IRB?**

○ Yes ● **No**

7. * Does the local principal investigator have a financial interest related to this research?

○ Yes ● **No**

8. Attach the protocol:

- Sponsor/Multicenter/Investigator-initiated protocol
- Coordinating Center supplement
- Emergency Use Consent/ Protocol/ FDA Form 3926
- Exempt Application form

	Document	Category	Date Modified	Document History
View	Developing a deliberate practice intervention(0.01)	IRB Protocol	12/3/2020	History

View: Pitt SF: Funding Sources (not integrated with Grants)

Funding Sources

1. * **Indicate all sources of support:**
   External funding

2. * **Identify each organization supplying funding for the study:**

Funding Source	Sponsor's Funding ID	Grants Office ID	Attachments	Pitt Awardee	Grant Recipient
National Institutes of Health				yes	

View: Pitt SF: Study Team Members

Study Team Members

1. * **Identify each person involved in the design, conduct, or reporting of the research (includes PI):**

Name	Roles	Department/School	Affiliation	Involved in Consent	Qualifications	Financial Interest
Robert Arnold	Co-investigator	U of Pgh | School of Medicine | Medicine	Pitt faculty	no	Dr. Arnold is a Distinguished Service Professor of Medicine and Director of the Section of Palliative Care and Medical Ethics in the School of Medici... view all	no
Jacqueline Barnes	Co-investigator	U of Pgh | School of Medicine | Critical Care Medicine	Pitt staff	no	Dr. Barnes is a qualitative researcher in the Department of Critical Care Medicine. She will be responsible for coding interviews and evaluating vide... view all	no
Jonathan Elmer	Co-investigator	U of Pgh | School of Medicine | Emergency Medicine	Pitt faculty	no	Dr. Elmer is focused on improving outcomes of cardiac arrest survivors. He works to achieve this goal through knowledge creation in three domains: ad... view all	no
Raquel Forsythe	Co-investigator	U of Pgh | School of Medicine | Surgery	Pitt faculty	no	Dr. Forsythe is a General Surgeon who practices Trauma Surgery, Elective and Emergency General Surgery and Critical Care Medicine at the Presbyterian... view all	no
Audrey Kenney	Co-investigator	U of Pgh | Associate Vice Chancellor of Human Resources | All Temps	Pitt staff	no	Ms. Kenney is a clinical research coordinator at the University of Pittsburgh in the Department of Critical Care Medicine	no

Name	Roles	Department/School	Affiliation	Involved in Consent	Qualifications	Financial Interest
Deepika Mohan	Principal Investigator	U of Pgh | School of Medicine | Critical Care Medicine	Pitt faculty	yes	Dr. Mohan is an associate professor of surgery and critical care medicine. She is the PI of the project, a role for which she is suited because of he... view all	no
Kimberly Rak	Co-investigator	U of Pgh | School of Medicine | Critical Care Medicine	Pitt staff	no	Dr. Rak is a qualitative researcher in the Department of Critical Care Medicine. She will be responsible for coding interviews and evaluating video t... view all	no
Mary Ryabik	Key Personnel / Support Staff	U of Pgh | School of Medicine | Critical Care Medicine	Pitt staff	no	Ms. Ryabik will be joining the team as the Project Manager. She will be helping to coordinate administrative tasks for the trial, including creating ... view all	no
Douglas White	Co-investigator	U of Pgh | School of Medicine | Critical Care Medicine	Pitt faculty	no	Dr. White is a Professor of Critical Care Medicine, who has completed a Master's degree on health services research methods. He has been/is a PI on 4... view all	no
Donald Yealy	Co-investigator	U of Pgh | School of Medicine | Emergency Medicine	Pitt faculty	no	Dr. Yealy is trained and experienced as a clinician/investigator, focusing on emergency medical care by examining pre-existing data and completing pr... view all	no

2. External team member information: (Address all study team members in item 1. above and leave this section blank)

Name	Description

Name	Description
There are no items to display	

3. **Have you,** Deepika Mohan**, verified that all members of the research team have the appropriate expertise, credentials, training, and if applicable, child clearances and/or hospital privileges to perform those research procedures that are their responsibility as outlined in the IRB application?**

* ● **Yes** ○ No

View: Pitt SF: Study Scope 8.1

Study Scope

Check all that apply

1. * Will this study actively recruit any of the following populations?

- ☐ Adults with impaired decision-making capacity
- ☐ Children (under the applicable law of the jurisdiction in which the research will be conducted (<18 years for PA))
- ☐ Children who are Wards of the State
- ☐ Employees of the University of Pittsburgh/UPMC
- ☐ Medical Students of University of Pittsburgh as primary research group
- ☐ Students of the University of Pittsburgh
- ☐ Neonates of uncertain viability
- ☐ Non-viable neonates
- ☐ Non-English speakers
- ☐ Nursing home patients in the state of Pennsylvania
- ☐ Pregnant women
- ☐ Prisoners
- ☑ N/A

2. * Will any Waivers be requested?

- ☐ Waiver/Alteration of Consent
- ☐ Waiver to Document Consent
- ☐ Waiver/Alteration of HIPAA
- ☐ Exception from consent for emergency research
- ☑ N/A

3. * Will this study involve any of the following?

- ☐ Specimens
- ☐ Honest Broker to provide data/specimens
- ☐ Return of Results to Subjects or Others
- ☐ Fetal tissue
- ☑ N/A

4. * Will Protected Health Information be collected?

- ☐ Pitt medical records
- ☐ UPMC medical records
- ☐ Other Institutions' medical records
- ☑ N/A

5. * Other Requests?

- ☐ Deception (if not Exempt, also requires Waiver/Alteration of Consent)
- ☐ Emergency Use / Single Patient Expanded Access (using FDA Form 3926)
- ☐ Placebo Arm

- [ ] Withdraw from usual care
- [x] N/A

6. * **Determining Scientific Review:**
Received External funding where scientific merit was established as a condition of funding

7. * **Has this study (or substantially similar study) been previously disapproved by the Pitt IRB or, to your knowledge, by any other IRB?**
○ Yes ● **No**

Review the HRPO policy, if participating in research at the VA Pittsburgh Healthcare System or using funding from the VA

8. * **Does the study use an approved drug or biologic, use an unapproved drug or biologic, or use a food or dietary supplement to prevent, diagnose, cure, treat, or mitigate a disease or condition?**
○ Yes ● **No**

9. * **Does the study evaluate the safety or effectiveness of a device (includes in-vitro laboratory assays)?**
○ Yes ● **No**

10. * **Is this application being submitted to convert an approved study from OSIRIS to PittPRO?** (Tip Sheet)
○ Yes ● **No**

11. * **Does your research protocol involve the evaluation or use of procedures that emit ionizing radiation and, after reviewing this HUSC guidance, does your research protocol require HUSC review? (If yes, upload the HUSC form in the Local Supporting Documents section). If you are unsure of review requirement, select yes.**
○ Yes ● **No**

View: Pitt SF: Research Sites

Research Sites

1. **Choose all sites that apply:**

   University of Pittsburgh

   * **Select the University of Pittsburgh sites where research will be conducted:**

   Main Campus – Pittsburgh

   List university owned off-campus research sites if applicable:

   N/A

2. **Describe the availability of resources and the adequacy of the facilities to conduct this study:**

   Resources to conduct the study include:
   1. The game we be refined using funds from an R21 grant, which will also be used to pay participants.
   2. The game will be hosted by the Department of Critical Care Medicine on a University of Pittsburgh server.
   3. The PI has a private office at the University of Pittsburgh to conduct data analysis in a secure and private location.

View: Pitt SF: Local Site Documents

Click **Continue** as this page was intentionally left blank.

View: Pitt SF: Recruitment Methods

Recruitment Methods

* **Will you be recruiting individuals for participation in this study?**
● **Yes** ○ No

1. * **Describe who will be recruiting individuals for participation for this study:**
Dr. Mohan will be running the recruitment.

2. * **Select all methods to be used for recruitment:**
Email/Listserv/Electronic Mailing List

3. * **Provide details on your recruitment methods:**
We plan to use participants from prior behavioral trials to facilitate snowball recruitment. We will contact prior participants, and will ask them to refer us to 1-2 colleagues who might be interested in participating in a research trial to assess physician decision making in trauma triage. Once we get the referrals, we will contact physicians, provide details of the trial, and then once receiving consent, will randomize them to either the intervention or the control arm of the pilot study. To supplement our numbers, we also plan to reach out to ERMI, a local professional staffing organization that provides emergency medicine physicians to non-trauma centers in the Pittsburgh region.

4. * **Describe all compensation/incentives offered to participants and timing of these offers:**
Physicians will receive $100/hour of time spent participating in the trial, a wage-based honorarium.

5. **Recruitment materials:** (attach all material to be seen or heard by subjects, including advertisements and scripts)

	Document	Category	Date Modified	Document History
View	Recruitment text_deliberate practice_Version_3.01.doc(2.02)	Recruitment Materials	12/14/2020	History

View: Pitt SF: Study Aims

Study Aims

1. * **Describe the purpose, specific aims, or objectives and state the hypotheses to be tested:**

Deliberate practice – goal-oriented training in the presence of a coach who can provide personalized, immediate feedback – has successfully improved performance across multiple domains, including sports, music, and combat. When used in conjunction with simulation to improve surgical skill, it has a large effect on educational outcomes. It has characteristics that make its application in this context potentially powerful (e.g. personalized feedback/relationship with coach increase engagement) but also potentially challenging (e.g. the diagnostic process does not lend itself easily to assessment). The objective of this R21 application is to test the feasibility of using deliberate practice to amplify the effect of our video game interventions. We will recruit a national sample of board-certified emergency physicians (n=30) to serve as trainees, with members of the team (n=3) serving as coaches. Trainee-coach dyads will meet for 30 minutes/week for 3 weeks, by video-conferencing, to play one of the existing video games and to use it to practice pattern recognition. We aim:

1. To assess the fidelity of intervention delivery. Approach: we will standardize coaching skill during an 'on-boarding session,' measure skill drift over the course of training sessions, and measure protocol adherence (primary outcome). Hypothesis: >90% of dyads will complete three training sessions.
2. To assess the potential effect size of the intervention. Approach: we will compare performance of trainees (n=30) with a control group of physicians (n=30) on a validated virtual simulation. Hypothesis: Trainees will make ≥25% fewer diagnostic errors than control physicians (large effect size).
3. To assess the acceptability of the intervention. Approach: we will conduct semi-structured debriefing interviews with trainees, assessing elements of the intervention that promote engagement.

This proposal will inform a future Stage III trial to compare the effect of different interventions on diagnostic error in trauma triage. If successful, this program of research will have an impact on patients by reducing the burden imposed by injury and by addressing the refractory problem of diagnostic error. It is novel conceptually in its effort to make heuristics a source of power, methodologically in its use of deliberate practice to improve diagnosis, and translationally in its use of video game technology. It is feasible because our multi-disciplinary team has clinical and behavioral science expertise, experience developing deliberate practice interventions, and a track record of successfully building video games that can transform physician behavior. It responds to two national research priorities: 1) improving the diagnostic process; 2) maintaining health and independent living among the aging.

2. * **Describe the relevant prior experience and gaps in current knowledge**

including preliminary data. Provide for the scientific or scholarly background for, rationale for, and significance of the research based on existing literature and how it will add to existing knowledge:
Diagnostic error is a particularly important problem for the 500,000 elderly patients who present to non-trauma centers every year after trauma. Emergency medicine physicians must rapidly categorize patients as having minor or severe injuries based on limited information, and decide whether or not to transfer the patients to a trauma center (triage). Observations from our group demonstrate that physicians rely on heuristics (intuitive judgments) to identify severely injured patients, which results in the systematic under-triage of the elderly. Under-triaged patients experience a 10-25% increase in mortality, loss of independence, and increased pain.

The absence of an effective means of recalibrating heuristics is a critical barrier to the improvement in outcomes for elderly patients with severe injuries. Behavioral scientists agree that people develop well-calibrated heuristics through an experience-feedback loop that hones pattern recognition and ensures the recognition of relevant contextual cues. Since replicating that loop has proven challenging outside of formal training programs (e.g. residency), most initiatives to reduce diagnostic error have focused on eliminating the use of heuristics. Unfortunately, they have had limited efficacy, probably because heuristics are essential to human cognition. The National Academy of Medicine therefore recently identified the development of a method of addressing the challenge posed by heuristics to be a major priority.

In prior work, we exploited insights from the behavioral science literature to develop novel interventions to recalibrate physician heuristics. Specifically, we identified surrogates for the experience-feedback loop in other domains (e.g. threat detection), and applied them to trauma triage (NIH Stage I behavioral intervention development). We delivered these interventions as video games to increase engagement and to facilitate dissemination. In pilot trials, physicians who played the games made fewer diagnostic errors on a validated virtual simulation compared to those who completed a gold-standard, text-based educational program, an effect that persisted to six month follow-up (Stage II development). These positive results occurred despite physicians reporting only moderate engagement with the interventions. Our overarching hypothesis is that by increasing engagement we can amplify the potency of the interventions. We therefore propose to refine the games before testing their efficacy in the real world (Stage III development).

View: Pitt SF: Study Design

Study Design

1. **Total number of subjects to be enrolled at this site (enter -1 for chart reviews, banking, registries):**

   60

2. **Describe and explain the study design:**

   We will modify the interface of one of our two games to ensure its' suitability as a training task. The three team members (Mohan, Elmer, Forsythe) who will serve as coaches will undergo training with our two deliberate practice experts (White, Arnold) to standardize their coaching skills. Next, we will recruit emergency physicians working at non-trauma centers around the country (N=60): 30 trainees and 30 passive controls. We will pair trainees with a coach, and will ask coach-trainee dyads to schedule 30-minute training sessions at their convenience, once per week for the three week period. At the completion of the three weeks, we will ask trainees to complete a semi-structured, debriefing interview and a virtual simulation to assess triage performance. We will ask passive controls to complete the same simulation within 3 weeks of enrollment.

3. **Describe the primary and secondary study endpoints:**

   Aim 1. To assess the fidelity of intervention delivery.
   Ha1: ≥90% of coach-trainee dyads will complete all three coaching sessions.
   We will summarize the proportion of coach-trainee dyads that complete all of the training sessions. To deem the intervention delivery strategy feasible, we will need to see ≥90% protocol adherence. We will also summarize the characteristics of game use by each trainee, including whether any of them use the game outside the deliberate practice sessions; assessments of coaching performance by independent raters and by trainees; assessments made by coaches of intervention receipt. Finally, we will perform a series of exploratory analyses using non-parametric tests (Fisher exact test, Wilcoxon-Mann Whitney, Kruskal Wallis, repeated measures regression models) to characterize the association between trainee characteristics and: a) protocol adherence; b) coaching skill (first session and overall); c) intervention receipt (first session and overall).

   Aim 2. To assess the potential effect size of the intervention.
   Ha2: We will detect a large difference in the diagnostic errors made by trainees and control physicians
   We will use the virtual simulation to assess physician diagnostic performance, and will use an intention-to-treat approach (i.e. all trainees regardless of whether they completed all the sessions will be asked to use the simulation). We will measure the proportion of severely injured patients appropriately triaged to a trauma center as per the American College of Surgeons guidelines.5 We will estimate both the group mean of diagnostic errors made by trainees and passive control physicians, and variability in the group-specific outcome. We will use a Students t-test to compare

the means of the two groups. To deem the intervention successful (and worth pursuing), we will need to detect a large difference (~25%) between the two groups. In exploratory analyses, we will also assess the effect of the intervention on heuristics by studying patterns of errors (as we have done previously). We will not test any mediators of efficacy because of the sample size.

Aim 3. To assess the acceptability of the intervention. We will summarize responses to the User Engagement Scale-Short Form. We will also use best practice methods to report qualitative results from the semi-structured interviews, focusing on participant perceptions of the effect of the intervention on engagement.

4. **Provide a description of the following study timelines:**

Duration of an individual subject's active participation:
3 months

Duration anticipated to enroll all subjects:
3 months

Estimated date for the investigator to complete this study (complete primary analyses):
3/30/2020

5. **List the inclusion criteria:**
Research subjects will be board-certified physicians who work in the ED of non-trauma centers in the US, and who manage primarily adult patients.

6. **List the exclusion criteria:**
We will exclude physicians who work exclusively at Level I/II trauma centers, who treat only children, or who work outside of the US.

7. **Will children or any gender, racial or ethnic subgroups be explicitly excluded from participation?**
● **Yes** ○ No

* **Identify the subgroups and provide a justification:**
We will exclude children since we are interested in physician decision making.

8. **Describe the power analysis used and cite your method of statistical analysis. If a power analysis is not possible, thoroughly justify the sample size required for the study, including appropriate literature citation (alternatively provide page reference in attached protocol):**
We will assess the feasibility of using deliberate practice to recalibrate physician heuristics including: barriers to implementation, fidelity, acceptability, the magnitude and variance of the treatment effect. We anticipate that ≥80% of physicians in both the trainee and control groups will complete the virtual simulation. Based on Cohen's power estimates for behavioral trials, with at least 20 physicians in each group, we can detect a large difference (0.80 standard deviation) in their performance, using a t-test, with power of 80% and alpha=0.1.73 Given the distribution of responses in the

past, this would manifest as a 25% difference in diagnostic error.

Please see page 72 of the attached protocol.

View: Pitt SF: Research Activities

Research Activities

1. * **Provide a detailed description of all research activities (including screening and follow-up procedures) that will be performed for the purpose of this research study. This description of activities should be complete and of sufficient detail to permit an assessment of associated risks.**

OVERVIEW

We will modify the interface of one of our two games to ensure its' suitability as a training task. The three team members (Mohan, Elmer, Forsythe) who will serve as coaches will undergo training with our two deliberate practice experts (White, Arnold) to standardize their coaching skills. Next, we will recruit emergency physicians working at non-trauma centers around the country (N=60): 30 trainees and 30 passive controls. We will pair trainees with a coach, and will ask coach-trainee dyads to schedule 30-minute training sessions at their convenience, once per week for the three week period. At the completion of the three weeks, we will ask trainees to complete a semi-structured, debriefing interview and a virtual simulation to assess triage performance. We will ask passive controls to complete the same simulation within 3 weeks of enrollment.

DESCRIPTION OF SHIFT

Shift is a puzzle video game developed to recalibrate physician heuristics in trauma triage. Players engage in analogical encoding – structured case comparison – to derive their own decision principles for triage. Specifically, players review cases and then identify contextual cues associated with the presentation of severely injured patients. Next, they synthesize those cues into simple, unifying triage principles. Theoretically, the process of derivation makes the principles memorable, and therefore more likely to become part of the physicians' heuristics. The game has approximately 2 hours of content, covers 10 triage decision principles, and allows repeated play of selected sections. It should lend itself well to deliberate practice because coaches can observe the contextual cues that physicians highlight during the process of case comparison and can provide personalized feedback on how they should integrate those cues into the pattern that they use when diagnosing trauma patients (i.e. recalibrate their heuristics).

DESCRIPTION OF VIRTUAL SIMULATION

We previously developed and validated a virtual simulation to study physician decision making in trauma triage. Physicians have to evaluate and to manage ten cases over 42 minutes, simulating a busy eight-hour ED shift. Each case includes a 2-D rendering of the patient, a chief complaint, vital signs which updates every 30 seconds, a history, and a written description of the physical exam. Physicians manage patients by selecting from a pre-specified list of 250 medications, studies, and procedures. The cases end when physicians either make a disposition decision (admit, discharge, transfer) or the patient dies. To measure diagnostic performance, we will collect information on decision making: diagnostic, therapeutic, and disposition decisions.

RESEARCH ACTIVITIES

At the time of enrollment, we will ask both trainees and passive control physicians to complete a questionnaire that surveys their personal characteristics (time required: 10 minutes). Trainees will be assigned a coach, and will be asked to schedule weekly 30-minute meetings for three weeks, at which time they will play a video game and will receive feedback on how to use best-practice triage decision principles (time required: 90 minutes). Trainees will also be asked to complete a short assessment of their coaches' performance after each session (time required: 5 min/session). After completion of the intervention, trainees will be scheduled to participate in a semi-structured debriefing interview (time required: 20 minutes). Both trainees and passive-control physicians will be asked to complete a virtual simulation that assesses their triage decision making (time required 42 minutes). Trainees will therefore spend 3 hours completing study tasks; passive control physicians will spend 1 hour. Trainees will receive an iPad with the video game pre-loaded at the time of enrollment. They will keep the iPad as their honorarium for participating in the study. Passive control physicians will receive a $100 gift card as a fixed, wage-based honorarium for participating in the study. We chose to provide the honorarium to minimize attrition and to ensure completion of study tasks. Based on our prior experience, the combination of framing participation as altruistic, e-mail reminders about completion of study tasks, and provision of a wage-based, fixed honorarium increases completion rates to as high as >80%. Given the costs of running a trial, and bias introduced by differential completion of study tasks, we believe that using an honorarium is warranted.

SOURCES OF DATA
Physician self-report:
1. Physician characteristics. We will ask all participants to respond to a questionnaire that asks about personal characteristics: demographics (e.g. age, sex, race), training (e.g. name of medical school, name of residency training program and fellowship [if applicable], year of board certification), professional experience (e.g. years in practice, number of shifts worked/month, number of patients treated/shift), and practice environment (e.g. trauma center designation of hospital; resource availability).
2. Trainee assessment of coaching performance. After each deliberate practice session, we will ask trainees to rate their coaches' skill along four domains (as in the Wisconsin Surgical Coaching Rubric), using a Likert scale.
3. Trainee assessment of the acceptability of the intervention. We will ask trainees to complete the User Engagement Scale (a 12-item instrument that assesses aesthetic appeal, attentional focus, perceived usability, and needs satisfaction). In addition, we will ask them to participate in a 20-minute debriefing interview in which we probe: 1) their engagement with the study tasks; 2) how the intervention affected their well-being (e.g. did they find the experience onerous or did it reinforce their intrinsic motivation); 3) barriers to implementation. Interviews will be audio-taped, transcribed, and reviewed.

Deliberate practice sessions. We will videotape all the coaching sessions between the trainee-coach dyads. We will review these video tapes to assess: 1) protocol adherence (i.e. we will calculate the proportion of dyads that complete all three training sessions); 2) coaching performance. Two independent raters will review the

video tapes using the Wisconsin Surgical Coaching Rubric to evaluate coaching skill drift. The Rubric scores performance along four domains (shares responsibility, uses questions/prompts to guide trainee in self-reflection, provides constructive feedback; guides goal setting), with individual components summed together at the end for an overall assessment of skill.

Shift.
The game uploads information on usage statistics (e.g. number of clicks, proportion of app used) to a database every time the iPad connects to a wireless network. We will use this added information to determine how trainees use the games, and to calculate the number who use the games outside the training sessions.

Virtual Simulation. We will ask physicians to complete a virtual simulation, and will collect information on their decisions: diagnostic (CT scan, x-rays, labs), therapeutic (medications, procedures, consults), and disposition (admit, discharge). The simulation will be available on a web-based browser, and will store responses on a secure server hosted on the University of Pittsburgh network.

2. Upload a copy of all materials used to collect data about subjects: (Attach all surveys, interview/focus group scripts, and data collection forms except for case report forms, SCID or KSADS):

	Document	Category	Date Modified	Document History
View	Demographics questionnaire.docx(0.01)	Data Collection	12/11/2020	History
View	WiSCOR Scoring Rubric(0.01)	Data Collection	12/3/2020	History
View	User Engagement Scale(0.01)	Data Collection	12/3/2020	History

3. * Will blood samples be obtained for research purposes?

○ Yes ● **No**

View: Pitt SF: Consent Process

Consent Process

Enter N/A in response to the following questions if a Waiver of Consent is requested for all research activities or if no subjects are being enrolled.

1. * **Indicate where the consent process will take place and at what point consent will be obtained:**
We will email physicians describing the study and asking if they are interested in participating. The email will include a link to a Qualtrics survey that hosts a more detailed description of the study, a consent form, and a demographics questionnaire. Once physicians consent to participate in the trial, they will be randomized to the intervention or passive control arm.

2. * **Describe the steps that will be taken to minimize coercion and undue influence, including assurance that there is sufficient time for subjects to make an informed decision:**
Physicians will be given three days to respond to the email, at which point they will receive a reminder. Physicians who do not respond within 1 week to the recruitment email will not be contacted further. To minimize coercion, the Qualtrics document will emphasize that participation is voluntary, and consent can be withdrawn at any point during the study.

3. **For studies that involve multiple visits, describe the process to ensure ongoing consent:**
The study will involve multiple rounds of coaching (three in total), for those assigned to the intervention arm. The study coordinator will ensure ongoing consent when scheduling followup coaching sessions.

4. * **Steps to be taken to ensure the subjects' understanding:**
We will ensure that all the documentation is piloted for clarity and readability prior to contacting trial participants. When recruiting physicians our email will specify that: 1) we are recruiting physicians for an NIH-funded study; 2) participation is voluntary; 3) they will receive no direct benefit from participation; 4) they will receive an honorarium for their time; 5) participation will require 3 hours of time, distributed over 3 weeks; 6) they may withdraw from the study at any time. We will also provide contact information for the PI, which they can use to gain further details about the trial. During the first coaching session and again during the debriefing interview, study personnel will confirm participant's understanding of the study and the requirements.

5. * **Are you requesting an exception to the IRB policy related to the informed consent process:**
○ Yes ● **No**

View: Pitt SF: Consent Forms

Consent Forms

1. **Consent Forms:**

	Document	Category	Date Modified	Document History
View	consent_deliberate practice_Version_0.03.docx(0.03)	Consent Form	12/14/2020	History

Refer to the following templates and instructional documents:

- Guidance - Consent Wording
- Template - Consent Document - Short Form
- HRP-090 - SOP - Informed Consent Process for Research
- HRP-091 - SOP - Written Documentation of Consent

View: Pitt SF: Electronic Data Management

Electronic Data Management

1. * **Will only anonymous data be collected (select NO if identifiers will be recorded at anytime during the conduct of the study)?**
○ Yes ● **No**

Select all identifiers to be collected during any phase of the research including screening:

Name:	☑	Internet Protocol (IP) Address:	☐
E-mail address:	☑	Web Universal Resource Locators (URLs):	☐
Social security #:	☐	Social security # (for Vincent payment only):	☐
Phone/Fax #:	☑	Full face photo images or comparable images:	☐
Account #:	☐	Health plan beneficiary #:	☐
Medical record #:	☐	Device identifiers/serial numbers:	☐
Certificate/license #:	☐	Vehicle identifiers/serial #/license plate #:	☐
		Biometric identifiers, finger and voice prints:	☐

* **a: Will you be collecting any of the following location data: geographic subdivisions smaller than a State such as street address, city, county, precinct, zip, geocodes, etc.?** ● **Yes** ○ **No**

* **b: Will you be collecting any date information such as birth date, death, admission, discharge, date of surgery/service?** ● **Yes** ○ **No**

c: List any other unique identifying numbers, characteristics or codes related to an individual that are to be collected:

d: Will you be collecting any data subject to the General Data Protection Regulation (GDPR)? ○ **Yes** ● **No**

* **For ALL identifiable data collected, will you be coding the data by removing the identifiers and assigning a unique study ID/code to protect the identity of the participant?** ● **Yes** ○ **No**

* **Will the data be HIPAA de-identified?** ● **Yes** ○ **No**

* **Briefly describe your plan to store coded data separately from the identifiable data:**
We will assign trial participants an identifier at the time of randomization. We will use the identifier when storing and analyzing all study data. The linkage file will be stored on the HSRDC virtual desktop.

2. * **During this study, will restricted data as defined by the University's Data Risk Classification matrix (https://www.technology.pitt.edu/security/data-risk-classification-and-compliance) be processed, stored, or transmitted?**
○ Yes ○ No

3. * **During this study, will sensitive data (https://www.hrpo.pitt.edu/electronic-data-security) be collected where disclosure of identifying information could have adverse consequences for subjects or damage their financial standing,**

employability, insurability, educational advancement, reputation or place them at risk for criminal or civil liability?

○ Yes ● **No**

4. * Select all locations where data will be stored or accessed (including e.g., personal / employer laptop or desktop):

	Storage Device	Description	Identifiable Data	Sensitive Data	De-Identified/Anonymous Data
View	Server: Pitt Department Managed Server	We will store all the trial data on the HSRDC desktop.	yes	no	yes
View	Server: Pitt Department Managed Server	We will store all the video recordings on the HSRDC desktop	yes	no	yes
View	Server: Pitt Department Managed Server	The virtual simulation will upload information to a website hosted by the Department of Critical Care Medicine.	no	no	yes

5. * Select all technologies being used to collect data or interact with subjects:

Electronic audio, photographic, or video recording or conferencing

Web-based site, survey, or other tool

6. * Video, Audio, Images – identify all uses of video, audio, photography, etc. to be used to collect data during any phase of the research:

	name	Identifiable
View	Video recording	yes

7. * Web Based Technologies – identify all web based technologies to be used to collect data during any phase of the research:

	name	Identifiable
View	Pitt Licensed Qualtrics	
View	Website	no

View: Pitt SF: Data Safety and Monitoring

Data Safety and Monitoring

1. * **Describe your plan to periodically evaluate the data collected regarding both harms and benefits to determine whether subjects remain safe. The plan might include establishing a data monitoring committee and a plan for reporting data monitoring committee findings to the IRB and the sponsor:**
The research team will meet weekly during data collection and then monthly for the remainder of the study period to evaluate data collection. During team meetings, we will review study protocols to ensure that no harms have occurred and that the benefits are as expected. If either study personnel or the coaches believe that an adverse event has occurred during data collection, they will report that information to the PI who will take responsibility for transmitting the information to the IRB and to the sponsor within 72 hours of notification.

2. * **Describe your plan for sharing data and/or specimens:**
To support the translation of research results into policy practice, manuscripts describing research results will be drafted and submitted in a timely manner for publication in widely circulated peer-reviewed journals. Dr. Mohan will also present interim and final results at relevant academic and non-academic conferences. Raw data and derived datasets will be made available to external investigators and the public on a case-by-case basis, to be approved by the PI, Dr. Mohan, and in accordance with institutional, HIPAA, state and federal regulations. A data-sharing agreement may be instituted, depending upon the data to be shared. All data that is shared will be de-identified to protect participant privacy and confidentiality. Data and datasets will be retained and available to share for at least three years following completion of the project, in accordance with NIH regulations. The research team will track and report on the use, dissemination and sharing of all data and datasets and assist the PI with administration of data-sharing agreements as necessary.

3. **If any research data is collected, stored, or shared in a paper format, address what precautions will be used to maintain the confidentiality of the data:**
N/A

View: Pitt SF: Risk and Benefits

Risk and Benefits

1. * **Enter all reasonably foreseeable risks, discomforts, hazards, or inconveniences to the subjects related to subjects' participation in the research:**

View

Research Activity	Coaching/completion of simulation/completion of questionnaires
Common Risks	Breach of privacy
Infrequent Risks	None
Other Risks	*No Value Entered*

2. * **Describe the steps that will be taken to prevent or to minimize risks:**

Every effort will be made to minimize risk. The investigators and study staff will achieve certification as required by the IRB at the University of Pittsburgh. All physicians will be assigned a study identifier at the time of enrollment, and all data associated with that physician will use that identifier. Information linking the data codes with subject identities will be stored separately from the recorded data. At no time will we reveal subject identities in any description or publication of the research for scientific purposes.

3. **Financial risks - will the subject or insurer be charged for any research required procedures?**

○ Yes ● **No**

4. **Describe the steps that will be taken to protect subjects' privacy:**

To protect participants' privacy we will take the following steps:
1. Coaching sessions and debriefing interviews will be conducted in private rooms.
2. We will not collect any sensitive information, since none is required for completion of the study aims.
3. All study subjects will be assigned unique study identifiers that will appear on all data collection instruments, documents, and files used in the statistical analysis and manuscript preparation.
4. Only limited team members will have access to personal information needed for tracking and informed consent. No personal information concerning study participants will be released without their written consent.

5. **What steps will be taken in the event that a clinically significant, unexpected disease or condition is identified during the conduct of the study:**

There are no foreseeable risks of identifying an unexpected disease since the objective of this project is to educate physicians.

6. **Describe the potential benefit that individual subjects may experience from taking part in the research or indicate if there is no direct benefit. Do not**

include benefits to society or others:

Coaching has the potential to improve physicians' performance and enhance their skill set.

7. **Do you anticipate any circumstances under which subjects might be withdrawn from the research without their consent?**

○ Yes ● **No**

8. **Describe procedures that will be followed when subjects withdraw from the research, including partial withdrawal from procedures with continued data collection and data already collected:**

If physicians withdraw from the study after partial completion of the study tasks, we will analyze their residual data. In particular, understanding reasons for their withdrawal are extremely important for achieving the study objectives (assessing the feasibility of using deliberate practice interventions).

View: Pitt SF: Conflict of Interest Ver2

Conflict of Interest

Institutional Financial Interests

1. * **To the best of your knowledge, has the University of Pittsburgh optioned or licensed technology that will be tested or evaluated in this research?**
   Yes No

View: Pitt SF: Ancillary Reviews

Ancillary Reviews

1. **Ancillary reviews or notifications selected below are required based on previous answers to questions. If a selection is incorrect, return to the appropriate page and adjust the answers to questions on that page:**

- [ ] Conflict of Interest (**COI**)
- [ ] Clinical and Translational Research Center (**CTRC**)
- [x] Data Security
- [ ] Honest Broker
- [ ] UPMC Investigational Drug Service
- [ ] Pitt Medical School Review
- [ ] Pitt+Me
- [ ] IND & IDE Support(**IIS**)
- [ ] Radioactive Drug Research Committee (**RDRC**)(study involves the evaluation or use of procedures that emit ionizing radiation)
- [ ] ORP Business **Manager** (required for industry sponsored studies)
- [ ] Religious Directives
- [ ] Scientific Review
- [ ] Health Record Research Request (**R3**) (required if using UPMC clinical data and authorization for other UPMC data sources for research)
- [ ] UPMC Office of Sponsored Programs and Research **Support** (using UPMC facilities and/or UPMC patients during the conduct of the study)

2. **Additional ancillary reviews the PI may choose to include as needed for the research:**

- [ ] Human Stem Cell Oversight (**hSCRO**)
- [ ] Institutional Biosafety Committee (**IBC**)(study involves deliberate transfer of recombinant or synthetic nucleic acid molecules)

View: Pitt SF: Clinical Trials

Good Clinical Practice (GCP) Training

1. * **Regardless of funding source, is this study a clinical trial (as defined by the NIH)?**
   ● **Yes** ○ No

ClinicalTrials.gov Information

Visit the University of Pittsburgh Office for ClinicalTrials.gov website or contact ctgov@pitt.edu for further information.

2. * **Was this study registered, or will it be registered, on ClinicalTrials.gov?**
   ● **Yes** ○ No

3. * **Is the University of Pittsburgh or UPMC the Sponsor Organization for this study record?**
   ● **Yes** ○ No

   * **Who will be the Responsible Party for this study record?**

   Principal Investigator of this IRB application

View: Pitt SF: Supporting Documents

Supporting Documents

1. **Attach any additional supporting documents not previously uploaded. Name the documents as you want them to appear in the approval letter:**

Document	Category	Date Modified	Document History

There are no items to display

View: Pitt Create

Add Storage Information

1. * **Select a Storage Type:**
   Server: Pitt Department Managed Server

2. **Description:**
   We will store all the trial data on the HSRDC desktop.

3. * **Will identifiable data be stored in this location?**
   ● **Yes** ○ No

4. * **Will sensitive data be stored in this location?**
   ○ Yes ● **No**

5. **Will de-Identified or anonymous data be stored in this location?**
   ● **Yes** ○ No

6. **Provide additional information as needed:**

View: Pitt Create

Add Storage Information

1. * **Select a Storage Type:**
   Server: Pitt Department Managed Server

2. **Description:**
   We will store all the video recordings on the HSRDC desktop

3. * **Will identifiable data be stored in this location?**
   ● **Yes** ○ No

4. * **Will sensitive data be stored in this location?**
   ○ Yes ● **No**

5. **Will de-Identified or anonymous data be stored in this location?**
   ● **Yes** ○ No

6. **Provide additional information as needed:**
   We plan to video tape Zoom conferences between study team members and trial participants - including game play of an intervention and debriefing interviews. The videos will be uploaded from study devices to the HSRDC desktop and then wiped from the devices. These videos will include physician identifiers (e.g. names, voices, faces) but no sensitive information.

View: Pitt Create

Add Storage Information

1. * **Select a Storage Type:**
   Server: Pitt Department Managed Server

2. **Description:**
   The virtual simulation will upload information to a website hosted by the Department of Critical Care Medicine.

3. * **Will identifiable data be stored in this location?**
   ○ Yes ● **No**

4. * **Will sensitive data be stored in this location?**
   ○ Yes ● **No**

5. **Will de-Identified or anonymous data be stored in this location?**
   ● **Yes** ○ No

6. **Provide additional information as needed:**
   Physicians will log into the website hosting the virtual simulation using only their trial identifier. No personal information will be recorded on this website.

View: Pitt Risk

Risk

1. * **Research Activity:**
   Coaching/completion of simulation/completion of questionnaires

2. **Common Risks:**
   Breach of privacy

3. **Infrequent Risks:**
   None

4. **Other Risks:**
