# Supplementary material for: Testing a Novel Deliberate Practice Intervention to Improve Diagnostic Reasoning in Trauma Triage: A Pilot Randomized Clinical Trial
Source: JAMA Netw Open. 2023 May 17;6(5):e2313569. doi: 10.1001/jamanetworkopen.2023.13569 (PMC10193186; doi:10.1001/jamanetworkopen.2023.13569)
Supplement: Supplement 3. — Data Sharing Statement [file jamanetwopen-e2313569-s003.pdf]

Data Sharing Statement

Mohan. Testing a Novel Deliberate Practice Intervention to Improve Diagnostic Reasoning in Trauma Triage. *JAMA Netw Open*. Published May 17, 2023.
doi:10.1001/jamanetworkopen.2023.13569

Data
Data available: Yes
Data types: Deidentified participant data
How to access data: mohand@upmc.edu
When available: With publication

Supporting Documents
Document types: None

Additional Information
Who can access the data: Researchers whose proposed use of the data has been approved
Types of analyses: Any purpose
Mechanisms of data availability: with a signed data access agreement
